# Intake of Soy Products and Other Foods and Gastric Cancer Risk: A Prospective Study

**DOI:** 10.2188/jea.JE20120232

**Published:** 2013-09-05

**Authors:** Kwang-Pil Ko, Sue K. Park, Jae Jeong Yang, Seung Hyun Ma, Jin Gwack, Aesun Shin, YeonJu Kim, Daehee Kang, Soung-Hoon Chang, Hai-Rim Shin, Keun-Young Yoo

**Affiliations:** 1 Department of Preventive Medicine, Gachon University, Incheon, Korea; 2 Department of Preventive Medicine, Seoul National University College of Medicine, Seoul, Korea; 3 Seoul National University Cancer Research Institute, Seoul, Korea; 4 Department of Biomedical Science, Seoul National University Graduate School, Seoul, Korea; 5 Division of Epidemic Intelligence Service, Korea Centers for Disease Control and Prevention, Osong, Korea; 6 National Cancer Control Research Institute, National Cancer Center, Goyang, Korea; 7 Cancer Registration & Statistics Branch, National Cancer Control Institute, National Cancer Center, Goyang, Korea; 8 Epidemiology Program, University of Hawaii Cancer Center, Honolulu, Hawaii, USA; 9 Department of Preventive Medicine, Konkuk University, Chungju, Korea; 10 Noncommunicable Diseases and Health Promotion, World Health Organization Western Pacific Regional Office, Manila, Philippines

**Keywords:** soybean, dietary intake, gastric cancer

## Abstract

**Background:**

Gastric cancer, the most common cancer in the world, is affected by some foods or food groups. We examined the relationship between dietary intake and stomach cancer risk in the Korean Multi-Center Cancer Cohort (KMCC).

**Methods:**

The KMCC included 19 688 Korean men and women who were enrolled from 1993 to 2004. Of those subjects, 9724 completed a brief 14-food frequency questionnaire at baseline. Through record linkage with the Korean Central Cancer Registry and National Death Certificate databases, we documented 166 gastric cancer cases as of December 31, 2008. Cox proportional hazard models were used to estimate relative risks (RRs) and 95% CIs.

**Results:**

Frequent intake of soybean/tofu was significantly associated with reduced risk of gastric cancer, after adjustment for age, sex, cigarette smoking, body mass index, alcohol consumption, and area of residence (*P* for trend = 0.036). We found a significant inverse association between soybean/tofu intake and gastric cancer risk among women (RR = 0.41, 95% CI: 0.22–0.78). Men with a high soybean/tofu intake had a lower risk of gastric cancer, but the reduction was not statistically significant (RR = 0.77, 95% CI: 0.52–1.13). There was no interaction between soybean/tofu intake and cigarette smoking in relation to gastric cancer risk (*P* for interaction = 0.268).

**Conclusions:**

Frequent soybean/tofu intake was associated with lower risk of gastric cancer.

## INTRODUCTION

Gastric cancer is the most commonly diagnosed cancer in the world^1^ and is a major cancer in Korea.^2^ Therefore, prevention of gastric cancer is an important target for cancer control.

Previous epidemiologic studies suggested that dietary intake has an important role in the etiology of gastric cancer.^3^ Salty foods were found to increase gastric cancer risk^4^^,^^5^ by damaging the stomach lining and increasing formation of endogenous N-nitroso compounds that have a known carcinogenic effect on gastric mucosa.^6^ In addition, a high-salt diet induces gastric epithelial hyperplasia and facilitates *Helicobacter pylori* infection.^7^

Although only a few studies have assessed the association between soy consumption and gastric cancer risk, it has been suggested that high intake of soy foods decreases the risk of gastric cancer.^8^^,^^9^ Because soy foods contain isoflavones that have anti-inflammatory and anti-oxidant effects, they could prevent development of gastric cancer.^10^ Although soybean paste is a type of soy food, it contains high levels of salt and N-nitroso compounds, which form during fermentation. Thus, soybean paste intake could be a risk factor for gastric cancer.^11^ Because salt could mask the beneficial effect of soybeans on the association between isoflavone and gastric cancer, it is crucial in an analysis of soy intake to separate unfermented soy foods, such as soybeans/tofu, from fermented, high-salt soy foods, such as soybean paste.

Cigarette smoking is a risk factor for gastric cancer, due to its inflammatory and oxidative effects.^12^^,^^13^ Because soybeans/tofu and vegetables have anti-inflammatory and antioxidant effects,^10^ there may be a combined effect of soy/tofu, vegetables, and cigarette smoking on gastric cancer risk.

Although there have been a considerable number of studies on dietary intake and gastric cancer, most epidemiologic studies were case-control studies, which can be biased by dietary recall and selection. Although there are some prospective studies of dietary intake and gastric cancer risk, the foods or food groups that affect gastric cancer risk remain unclear.

The Korean Multi-Center Cancer Cohort (KMCC), which was designed to investigate the relationships between exposures and cancer risks, enrolled 19 688 subjects between 1993 and 2004. In the present prospective study, we examined the association between dietary intake and the risk of gastric cancer, with a focus on soy products, and assessed the combined effects of soybeans/tofu, vegetables, and cigarette smoking on gastric cancer risk in Korean adults.

## METHODS

### Study population and data collection

The Korean Multi-Center Cancer Cohort (KMCC) is a community-based prospective cohort study established in 4 urban and rural Korean areas (Haman, Chungju, Uljin, and Youngil) from 1993 through 2004.^14^ It consists of male and female voluntary participants and includes a questionnaire, anthropometric measurements, blood sampling, and spot urine sampling. Data on general lifestyle, physical activity, diet, reproductive factors, and agricultural exposures were obtained through interviews, using a structured questionnaire. The 9724 subjects with no gastric cancer completed the food frequency questionnaire until 2004. Anthropometric measurements and some clinical laboratory findings were also collected. Serum and buffy coat samples were stored at −70°C, and urine samples were stored at −20°C.

As of December 31, 2008, 166 cases of gastric cancer were identified through record linkage with the Central Cancer Registry and the National Death Certificate databases; 8 cases were identified by death certificate only among the 166 incident cases. Informed consent was obtained from participants at the interviews. The study protocol was approved by the institutional review boards of Seoul National University Hospital and the National Cancer Center of Korea.

### Dietary assessment

A self-administered food frequency questionnaire was used to monitor consumption of 14 food items among participants from Haman and Chungju, beginning in 1995. The 14 food items included vegetables, fruits, soy/tofu, soybean paste, seaweed, mushrooms, meat, dairy products, fresh fish, salted fish, eggs, instant food, fried food, and coffee. Soy/tofu includes unfermented soy foods, and soybean paste is a fermented soy food with a high salt content.

For each food item, participants were asked to report the average frequency of consumption, using 4 categories (almost never, 1–4 times/month, 1–4 times/week, ≥1 time/day).

### Statistical analysis

Data were analyzed using the Statistical Analysis System (SAS version 9.1, SAS Institute, Cary, NC, USA). The Cox proportional hazards model was used to determine relative risks (RRs) and 95% CIs for gastric cancer incidence, as compared with the “almost never” group, for each food intake-frequency category. The analysis was adjusted for age (continuous variable), sex, cigarette smoking, body mass index (BMI, [continuous variable]), alcohol drinking, and area of residence. Because the effect of isoflavones as a phytoestrogen differs between men and women, we assessed the association of soy food consumption with gastric cancer risk by sex. We also assessed the interaction of soy food intake and cigarette smoking in relation to gastric cancer risk, according to frequency of dietary intake and cigarette smoking status.

## RESULTS

The mean (SD) age of study subjects was 57.6 (11.2) years. Age ranged from 30 to 90 years. The mean (SD) follow-up period was 8.5 (3.7) years. The incidence of gastric cancer and characteristics of the study population are shown in Table 1. Among men and women, age significantly differed between those who did and did not develop gastric cancer. Both residence area and BMI marginally differed between men who developed gastric cancer and those who did not.

**Table 1.** Baseline characteristics of the study cohort

Variable	Men			Women		
	
	Incident cases (*n* = 116)	Non-cases (*n* = 3598)	*P* value	Incident cases (*n* = 50)	Non-cases (*n* = 5960)	*P* value
	*n* (%)	*n* (%)		*n* (%)	*n* (%)	
Age (years)			<0.001			0.002
<50	6 (5.2)	821 (22.8)		3 (6.0)	1573 (26.4)	
50–59	33 (28.5)	944 (26.2)		14 (28.0)	1666 (28.0)	
≥60	77 (66.4)	1833 (50.9)		33 (66.0)	2721 (45.6)	
Residence area			0.056			0.816
Chungju (urban)	52 (44.8)	1936 (53.8)		28 (56.0)	3435 (57.6)	
Haman (rural)	64 (55.2)	1662 (46.2)		22 (44.0)	2525 (42.4)	
Smoking status			0.193			0.754
Never-smoker	16 (13.8)	736 (20.5)		45 (90.0)	5406 (91.1)	
Ex-smoker	33 (28.5)	889 (24.8)		1 (2.0)	110 (1.8)	
Current smoker	67 (57.8)	1967 (54.8)		4 (8.0)	421 (7.1)	
Alcohol status			0.358			0.141
Never-drinker	26 (22.4)	957 (26.7)		39 (78.0)	4673 (78.9)	
Ex-drinker	17 (14.7)	398 (11.1)		3 (6.0)	122 (2.1)	
Current drinker	73 (62.9)	2228 (62.2)		8 (16.0)	1124 (19.0)	
Body mass index (kg/m^2^)			0.071			0.949
<23	67 (60.9)	1738 (50.1)		17 (36.2)	2144 (37.6)	
23–24.9	23 (20.9)	837 (24.2)		11 (23.4)	1386 (24.3)	
≥25	20 (18.2)	891 (25.7)		19 (40.4)	2174 (38.1)	

The associations of consumption of selected food items with gastric cancer risk are presented in Table 2. We found a significant inverse association between soy/tofu intake more than 3 to 4 times a week and gastric cancer risk (RR = 0.57, 95% CI: 0.34–0.98), after adjustment for potential confounders. In addition, frequent intake of fried food increased risk of gastric cancer, with a dose-response relationship (*P* for trend = 0.028). Increased intake of green vegetables decreased risk of gastric cancer, and increased intake of soybean paste increased risk of gastric cancer, although not significantly so.

**Table 2.** Relative risk (RR) and 95% CI of gastric cancer incidence for each food type assessed in the study cohort

Food items	Frequency of food intake							*P* for trend

	Almost never	1–4 times/month		1–4 times/week		≥1 time/day		
			
	Cases/person-years	Cases/person-years	RR^a^ (95% CI)	Cases/person-years	RR^a^ (95% CI)	Cases/person-years	RR^a^ (95% CI)	
Vegetables	5/1663	11/5196	0.80 (0.28–2.29)	31/16 286	0.66 (0.25–1.70)	105/51 794	0.68 (0.27–1.68)	0.484
Fruit	11/6008	43/21 110	1.12 (0.58–2.18)	83/36 363	1.42 (0.75–2.68)	29/18 898	1.10 (0.55–2.22)	0.592
Soybean/tofu	22/8670	79/33 032	0.92 (0.57–1.48)	37/24 963	0.57 (0.34–0.98)	27/15 455	0.68 (0.38–1.21)	0.036
Soybean paste	2/1870	7/3823	1.93 (0.40–9.31)	41/24 432	1.70 (0.41–7.06)	115/52 129	2.01 (0.52–8.50)	0.181
Seaweed	27/14 264	65/31 981	1.17 (0.75–1.84)	56/27 372	1.27 (0.80–2.03)	18/8682	1.50 (0.82–2.76)	0.170
Mushrooms	89/42 996	58/25 690	1.10 (0.79–1.54)	13/11 020	0.67 (0.37–1.22)	5/2350	1.15 (0.46–2.84)	0.641
Meat	19/13 332	100/44 530	1.40 (0.85–2.29)	43/22 471	1.05 (0.60–1.84)	4/2008	0.88 (0.30–2.60)	0.545
Dairy products	61/34 224	45/20 344	1.15 (0.78–1.70)	33/15 346	1.25 (0.82–1.92)	27/12 458	1.30 (0.83–2.06)	0.191
Eggs	58/28 063	64/32 114	0.97 (0.68–1.39)	31/18 268	0.85 (0.54–1.32)	12/3855	1.23 (0.65–2.31)	0.922
Fresh fish	39/20 897	76/35 496	1.12 (0.76–1.66)	43/23 639	0.95 (0.61–1.47)	7/2347	1.46 (0.65–3.28)	0.904
Salted fish	43/20 808	69/36 256	0.89 (0.60–1.32)	46/22 569	0.90 (0.58–1.39)	8/2712	1.24 (0.58–2.64)	0.990
Instant food	70/37 504	56/29 670	0.88 (0.61–1.25)	33/12 937	1.09 (0.71–1.67)	6/1886	1.31 (0.56–3.06)	0.655
Fried food	105/55 901	45/20 456	1.27 (0.90–1.81)	12/4871	1.51 (0.82–2.77)	4/1004	2.31 (0.85–6.30)	0.028
Coffee	57/33 024	35/11 333	1.58 (1.04–2.42)	27/10 676	1.35 (0.85–2.15)	46/27 317	0.94 (0.63–1.41)	0.756

^a^Adjusted for age, sex, cigarette smoking, body mass index, alcohol drinking, and area of residence.

The associations of consumption of selected food items with gastric cancer risk according to sex are presented in Table 3. Food frequencies were classified into low and high intakes by using the median. We found a significant inverse association between soybean/tofu intake and gastric cancer risk among women (RR = 0.41, 95% CI: 0.22–0.78). Among men, high soybean/tofu intake reduced gastric cancer risk, but not significantly so (RR = 0.77, 95% CI: 0.52–1.13).

**Table 3.** Relative risk (RR) and 95% CI of gastric cancer incidence for each food type, according to sex

Food items	Men			Women		
	
	Low intake (Cases/person-years)	High intake (Cases/person-years)	RR^a^ (95% CI)	Low intake (Cases/person-years)	High intake (Cases/person-years)	RR^a^ (95% CI)
Vegetables	34/8804	73/19 137	0.91 (0.60–1.49)	13/14 342	32/32 658	1.08 (0.55–2.13)
Fruit	39/10 135	77/20 413	1.04 (0.92–1.19)	15/16 984	35/34 848	1.11 (0.90–1.36)
Soybean/tofu	66/15 326	49/15 091	0.77 (0.52–1.13)	35/26 377	15/25 327	0.41 (0.22–0.78)
Soybean paste	35/10 822	80/19 677	1.06 (0.93–1.21)	15/19 303	35/32 452	1.10 (0.90–1.34)
Seaweed	65/18 413	51/12 094	1.08 (0.95–1.22)	27/27 833	23/23 960	1.01 (0.83–1.22)
Mushrooms	61/15 149	54/15 230	1.00 (0.89–1.14)	28/27 846	22/23 831	1.00 (0.83–1.22)
Meat	78/17 807	38/12 707	0.91 (0.80–1.03)	41/40 055	9/11 772	0.97 (0.76–1.24)
Dairy products	41/11 749	75/18 783	1.05 (0.92–1.19)	20/22 476	30/29 366	1.10 (0.91–1.33)
Eggs	34/8483	81/22 017	1.01 (0.89–1.16)	24/19 580	26/32 220	0.92 (0.76–1.11)
Fresh fish	78/19 193	37/11 311	0.96 (0.84–1.09)	37/37 200	13/14 675	1.02 (0.82–1.26)
Salted fish	74/19 650	42/10 862	1.02 (0.90–1.16)	38/37 415	12/14 419	0.97 (0.78–1.21)
Instant food	40/10 349	75/20 025	1.00 (0.88–1.14)	30/27 154	20/24 468	0.95 (0.79–1.16)
Fried food	73/20 049	43/10 430	1.10 (0.97–1.26)	32/35 852	18/15 901	1.14 (0.93–1.39)
Coffee	57/13 820	58/16 678	1.00 (0.88–1.13)	35/30 537	15/21 315	0.92 (0.75–1.13)

Low and high intake were determined by using the median intake for each food item.

^a^Adjusted for age, cigarette smoking, body mass index, alcohol drinking, and area of residence.

The combined effect of diet and cigarette smoking on gastric cancer risk among men is shown in Table 4. Among smokers, soybean/tofu intake reduced gastric cancer risk, but no significantly so (RR = 0.63, 95% CI: 0.36–1.09). Among nonsmokers, soybean/tofu intake was not associated with gastric cancer risk (RR = 0.82, 95% CI: 0.43–1.57). There was no interaction between soybean/tofu intake and cigarette smoking in relation to gastric cancer risk (*P* for interaction = 0.268). There was no interaction of soybean paste, vegetables, or fried food with cigarette smoking in relation to gastric cancer risk.

**Table 4.** Association of soy foods, vegetables, soybean paste, and fried food consumption with gastric cancer risk among men, according to current smoking status

Food items	Smokers			Nonsmokers			*P* for interaction
	
	No. of Cases	Person-years	RR^a^ (95% CI)	No. of Cases	Person-years	RR^a^ (95% CI)	
Soybean/tofu							0.268
Low intake	43	11 897	1.00	23	3409	1.00	
High intake	23	12 025	0.63 (0.36–1.09)	26	3044	0.82 (0.43–1.57)	
Soybean paste							0.608
Low intake	21	8369	1.00	14	2425	1.00	
High intake	45	15 633	0.99 (0.58–1.68)	35	4030	1.31 (0.69–2.48)	
Vegetables							
Low intake	19	7122	1.00	15	1666	1.00	0.992
High intake	45	14 726	0.83 (0.48–1.45)	28	4385	1.02 (0.52–1.99)	
Fried food							
Low intake	43	15 943	1.00	30	4081	1.00	0.983
High intake	24	8098	1.35 (0.79–2.30)	19	2314	1.18 (0.64–2.20)	

Low and high intake were determined by using the median intake for each food item.

^a^Adjusted for age, body mass index, alcohol drinking, and area of residence.

Because the smoking rate among women was low, we could not assess the interaction between soybean/tofu intake and cigarette smoking in relation to gastric cancer risk.

## DISCUSSION

We studied whether dietary habits affected gastric cancer risk. Our results showed that dietary soybean/tofu intake decreased the risk of gastric cancer and that the beneficial effect of soybean/tofu was stronger among women than among men. However, there was no interaction between soybean/tofu intake and cigarette smoking, with respect to gastric cancer risk.

Epidemiologic studies have only rarely investigated the relationship between soy product intake and gastric cancer. In a case-control study in Korea, a group with a high intake of soy products had a lower risk of gastric cancer than a group with a low intake of soy products.^15^ In a case-control study of Japanese adults, both soybean paste and tofu appeared to be related to gastric cancer, but the results were not statistically significant.^16^ However, in a cohort study of Japanese subjects, soybean paste and tofu did not affect gastric cancer risk.^17^ In a case-control study of Chinese adults, fermented soy products increased the risk of gastric cancer, but there was no link between unfermented soy products and gastric cancer risk.^18^

Most previous investigations were case-control studies and failed to show consistent results. The present study is a prospective cohort study and used dietary information from people without gastric cancer. As compared with case-control studies, our results are less likely to be affected by bias regarding dietary information. A recent prospective study showed that soy product intake decreased gastric cancer mortality.^9^

Although the results of previous studies were inconsistent, it is possible to posit general mechanisms by which soybean products could protect against gastric cancer. The benefits of soybean intake could be due to isoflavones such as genistein and daidzein, which have anti-inflammatory and anti-oxidative effects mediated by inhibition of protein-tyrosine kinases, DNA topoisomerases, and ribosomal S6 kinase.^10^^,^^19^ In addition, evidence from several experimental studies suggests that soy product intake decreases gastric cancer risk. In those studies, apoptosis of human gastric carcinoma cells was induced by genistein^20^ and *H pylori* growth was clearly inhibited by genistein.^21^

However, epidemiologic studies have shown an association between increased risk of gastric cancer and high intake of fermented soybean paste.^22^^,^^23^ Fermented soybean pastes contain high levels of salt and nitrate, due to fermentation.^11^ Nitrate is endogenously reduced to nitrite by oral bacteria, leading to formation of carcinogenic N-nitroso compounds, which can induce gastric carcinogenesis.^24^ Fermented soybean pastes contain considerable salt and activate *H pylori* colonization and development of atrophic gastritis.^7^ The differing associations of soybean/tofu and soybean paste with gastric cancer are consistent with those noted in a recent meta-analysis.^25^

In our study, the beneficial effect of soybean/tofu on gastric cancer was more prominent among women than among men. We hypothesize that female sex hormones might protect against gastric cancer by increasing apoptosis and regulating growth and clonal expansion of human gastric cancer cells.^26^^,^^27^ A recent meta-analysis showed that longer exposure to either endogenous or exogenous estrogen decreased the risk of gastric cancer.^28^ Because soybean products contain isoflavones—which are a type of phytoestrogen and act as estrogen agonists or antagonists, depending on endocrine estrogenic levels—the beneficial effect of soybean/tofu may differ by sex.

Soybean products have anti-inflammatory and antioxidant effects, while smoking has inflammatory and oxidative effects.^10^^,^^13^ Antioxidant deficiency, either due to dietary deficiencies or the increase in antioxidant requirements caused by smoking, may be a factor in gastric cancer development.^29^ In our study, although the beneficial effects of soybean/tofu for gastric cancer were more prominent among smokers than among nonsmokers, intake of soybean/tofu and smoking did not synergistically affect gastric cancer risk.

Previous studies found that vegetable and fruit intake decreased the risk of gastric cancer. Nevertheless, a meta-analysis that assessed the relationship between vegetable intake and gastric cancer among a prospective cohort found no association.^30^ Riboli et al conducted a meta-analysis of cohort studies and showed that vegetable and fruit intake lowered the incidence of gastric cancer; however, the strength of the association was lower than that observed in a meta-analysis of case-control studies.^31^ A possible reason why the strength of the association was higher for case-control studies than for cohort studies is that recall bias and selection bias are more likely in case-control studies.^31^ Another possible explanation for inaccurate measurements of exposures is that the limited variability of dietary intake was underestimated.^31^

In our study, there was no evidence that vegetable and fruit intake decreased the risk of gastric cancer. Koreans tend to eat cooked vegetables with salt instead of fresh vegetables, which is not the case among white populations, so it is difficult to draw conclusions regarding the beneficial effects of vegetable intake, due to limited information on cooking methods.

The study of the association between fried food consumption and gastric cancer risk requires more research. Frying at high temperatures produces potential carcinogens such as heterocyclic amines and acrylamide.^32^ A recent case-control study in northwest Iran reported a positive association between fried food consumption and gastric cancer risk.^33^

Our study had several limitations. First, as in other studies, the food frequency questionnaire may not have accurately measured dietary intake. In addition, only information on frequency of food intake was available, which made it difficult to calculate total food intakes in our study. In our previous nested case-control study, however, we measured serum isoflavone concentrations and found an inverse association between isoflavone concentration and gastric cancer.^34^ The results of the present study using a food-frequency questionnaire are consistent with those of a previous study using isoflavone biomarkers. The correlation coefficient between frequency of soybean intake and serum isoflavone concentration was 0.10. Second, there was no information on cooking methods. In some studies, different cooking methods, such as broiling and pan-frying, resulted in varying rates of gastric cancer risk, and pickled vegetables were associated with a higher gastric cancer risk than were unpickled vegetables.^23^ In the present analysis, however, we could not distinguish pickled from unpickled vegetables and salted from fresh fish. In our study, salted and fresh fish were categorized by salt level before cooking. However, because Koreans add considerable salt to fresh fish during cooking, the association of fresh fish with gastric cancer might be similar to that observed for salted fish. Third, the recruitment period was rather long, and the effects of food consumption could vary between generations. However, we conducted an analysis stratified by recruitment period (early vs late recruitment) and found no difference in the results (*P* for heterogeneity > 0.05). Fourth, we had no information on *H pylori* infection, which is an important factor in gastric cancer among Koreans.

Nevertheless, our study had several strengths. The cohort study design allowed us to prospectively investigate the association between dietary intake and gastric cancer among Koreans, and we analyzed associations between gastric cancer and foods that have not been sufficiently researched, such as mushrooms and seaweed. Our study subjects were Asians who consumed many soy and soybean products. Average daily isoflavone intake is 0.6 to 1.6 mg and 35 mg among Western and Asian populations, respectively, which suggests that epidemiologic studies of soy product intake among Asian populations would be more effective.^35^

In conclusion, the findings of this cohort study suggest that frequent consumption of soy products reduces the risk of stomach cancer. To obtain further reliable evidence on the relationship between soy products and gastric cancer, it will be necessary to understand dietary habits and design studies with efficient research methods.
